# A Rare Case of Giant Uterine Leiomyomata: A Difficult Diagnosis Accompanied by Surgical Difficulties

**DOI:** 10.7759/cureus.78764

**Published:** 2025-02-09

**Authors:** Efthymia Thanasa, Anna Thanasa, Emmanouil M Xydias, Evangelos Kamaretsos, Gerasimos Kontogeorgis, Ioannis Paraoulakis, Apostolos C Ziogas, Ioannis Thanasas

**Affiliations:** 1 Department of Health Sciences, Medical School, Aristotle University of Thessaloniki, Thessaloniki, GRC; 2 Department of Obstetrics and Gynecology, EmbryoClinic IVF, Thessaloniki, GRC; 3 Department of Obstetrics and Gynecology, University General Hospital "Attikon" Medical School, National and Kapodistrian University of Athens, Trikala, GRC; 4 Department of Obstetrics and Gynecology, General Hospital of Trikala, Trikala, GRC; 5 Department of Obstetrics and Gynecology, University of Thessaly, Larissa, GRC

**Keywords:** case report, giant leiomyoma, preoperative diagnosis, surgical treatment, symptoms, uterus

## Abstract

Leiomyomas, also known as fibroids, are a group of benign smooth muscle tumors commonly present in premenopausal women. Giant uterine leiomyomas are rare. It is described as giant when it weighs 11.4 kg. This report concerns a 45-year-old, asymptomatic woman of reproductive age who presented for her first gynecological consultation at the clinic of Trikala General Hospital, Greece, for a routine smear and gynecological examination. A large, painless abdominal mass was palpated during the clinical exam, reaching the level of the xiphoid process. Imaging confirmed the clinical diagnosis of a myomatous uterus, and surgical intervention via laparotomy was decided. Intraoperatively, a giant uterine leiomyoma was identified, leading to an abdominal total hysterectomy with bilateral salpingectomy and oophorectomy. A histological examination confirmed the diagnosis. After a five-day hospitalization with an uneventful postoperative recovery, the patient was discharged. This rare case emphasizes the uncommon presentation of a giant uterine leiomyoma in an asymptomatic reproductive-age patient, highlighting the diagnostic and surgical challenges associated with managing such cases.

## Introduction

Uterine fibroids, also known as leiomyomas, are benign monoclonal tumors originating from smooth muscle cells of the myometrium, intertwined with connective tissue bundles [1]. They are the most common benign pelvic tumors in women aged 30-44 years. Among women over 35, the prevalence is estimated at 40-60%, and in some populations, the overall prevalence in reproductive-age women may reach up to 70% [2,3]. The etiology of uterine leiomyomas is highly complex and remains poorly understood. A lack of knowledge about the molecular mechanisms initiating or promoting their pathogenesis and the absence of preclinical models replicating the molecular environment hinder effective long-term treatment [4]. Uterine fibroids can be asymptomatic or associated with symptoms such as abnormal uterine bleeding, anemia, pelvic pain, or pressure symptoms depending on the tumor’s location and size [5]. Based on their anatomical location relative to the uterus, fibroids are classified as intramural, subserosal, submucosal, or pendunculated [6]. Fibroid size can range from less than 1 cm to massive uterine fibroids [7]. In extremely rare cases, a fibroid can develop into a giant uterine leiomyoma, defined as a fibroid weighing 11.34 kg or more [8]. The first case of a giant uterine leiomyoma was described by Jonas and Masterson in 1977 [9]. In our case, after performing an abdominal total hysterectomy, the uterus, along with fibroids, fallopian tubes, and ovaries, was removed, measuring 35 × 26 × 22 cm and weighing 9.8 kg. Although the fibroid’s weight did not exceed 11.34 kg, the patient can be included among those treated for a giant uterine leiomyoma.

This rare case underscores the unusual occurrence of a giant uterine leiomyoma in a reproductive-age patient who was asymptomatic and unaware of the tumor’s presence. The report highlights common and atypical symptoms of giant uterine leiomyomas, emphasizing the diagnostic and surgical challenges associated with managing these patients.

## Case presentation

This case involves a 45-year-old reproductive-age woman who visited the gynecological clinic at the General Hospital of Trikala, Greece, for a Pap smear and routine gynecological examination. The patient was of low socioeconomic status, had mild intellectual disability of unknown etiology, was nulliparous, had a normal menstrual cycle, and reported her last period about a week prior. Menstrual duration was five days with normal blood flow. Her medical and family history was unremarkable, with no known gynecological conditions or systemic symptoms. The patient and her surroundings confirmed that it was her first visit ever to a gynecologist. Her BMI was 27.

**Figure 1:**
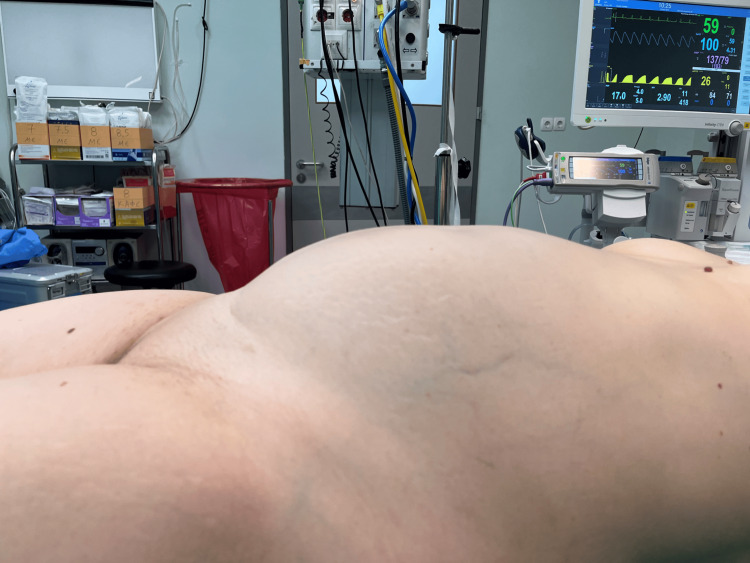
Patient in a supine position on the operating table before the start of surgery The presence of an abdominal mass is evident, with its upper boundary clearly delineated at the level of the xiphoid process.

Clinical examination revealed a painless, large abdominal mass palpable up to 2 cm below the xiphoid process (Figure 1).

**Figure 2:**
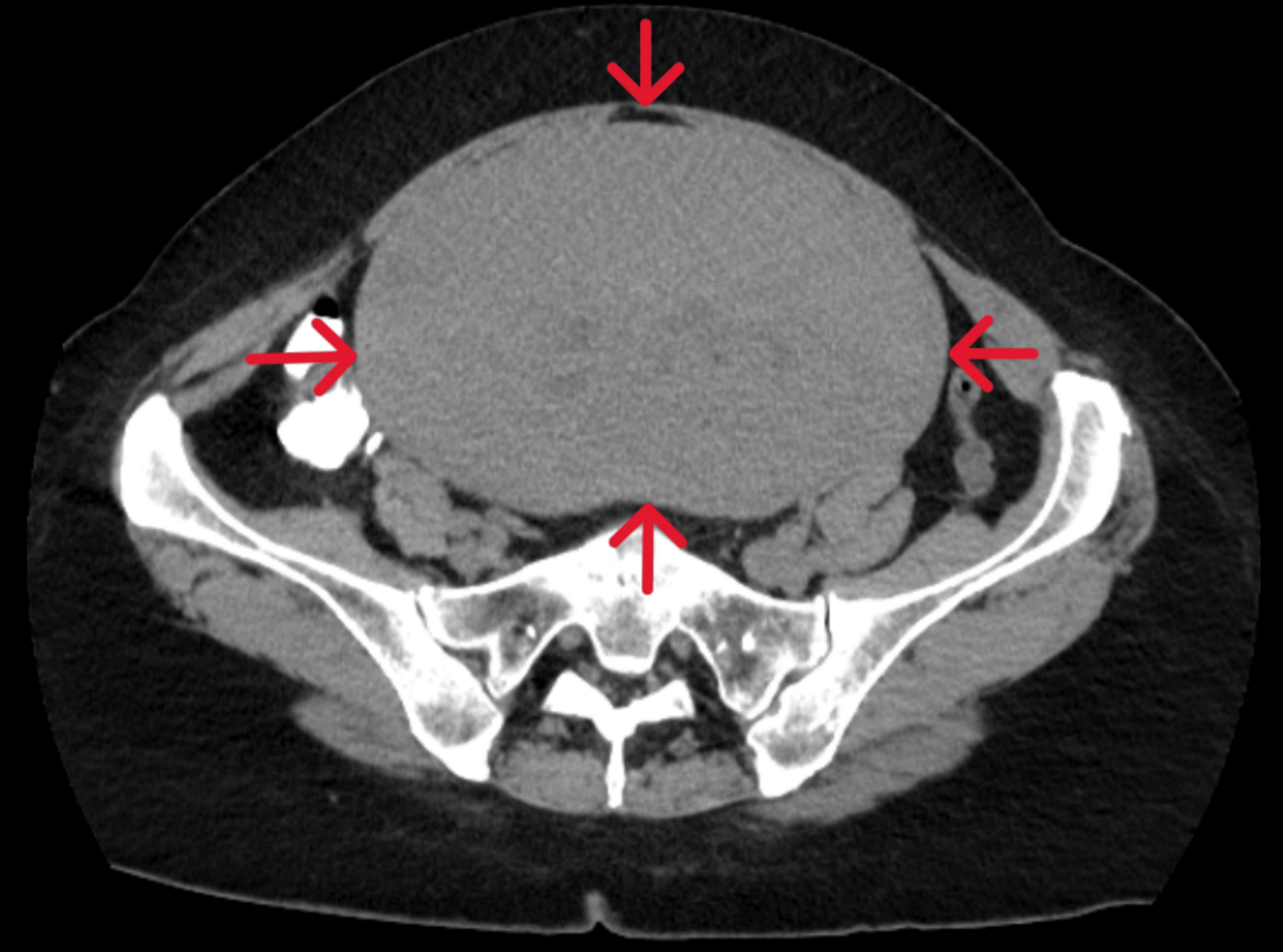
CT imaging of a giant uterine leiomyoma The scan reveals a well-defined pelvic mass of significant size with solid characteristics (red arrows), occupying the majority of the peritoneal cavity.

Abdominal and transvaginal ultrasounds were inconclusive. A CT scan revealed a solid, distinct, echogenic pelvic mass, measuring 37 × 27 × 23 cm, occupying most of the abdomen (Figure 2).

MRI of the pelvis was not performed, as the earliest available scheduled appointment at our hospital was in three months. Preoperative lab findings included normal hematocrit (40.3%), hemoglobin (13.4 g/dl), platelet count (215.000/mL), and white blood cell count (9.100/μL) with neutrophils 69.5%. Tumor markers were negative except for cancer antigen 125. The pregnancy test was negative. The biochemical analysis and the general urinalysis revealed no pathological findings (Table 1).

**Table 1:** Laboratory test results from the preoperative evaluation to the patient’s discharge from the clinic APTT, activated partial thromboplastin time; CA125, cancer antigen 125; CA15-3, cancer antigen 15-3; CA19-9, cancer antigen 19-9; CEA, carcinoembryonic antigen; Cr, creatinine; FIB, fibrinogen; Glu, glucose; Hb, hemoglobin; Ht, hematocrit; INR, international normalized ratio; K⁺, potassium; Na⁺, sodium; NEUT, neutrophils; PLT, platelets; WBC, white blood cells

Laboratory tests	Preoperative values	First postoperative day after surgery	Third postoperative day after surgery	Laboratory reference values
Ht	40.30%	30.10%	27.10%	37.7-49.7%
Hb	13.4 gr/dl	10.1 gr/dl	8.9 gr/dl	11.8-17.8 gr/dl
PLT	215 × 10³/ml	189 × 10³/ml	201 × 10³/ml	150-350 × 10³/ml
WBC	9.1 × 10³/ml	15.6 × 10³/ml	8.2 × 10³/ml	4-10.8 × 10³/ml
NEUT	69.50%	88.50%	66.50%	40-75%
APTT	26.2 seconds	28.6 seconds	27.4 seconds	24.0-35.0 seconds
INR	0.91	0.98	0.94	0.8-1.2
FIB	295 mg/dl	237 mg/dl	241 mg/dl	200-400 mg/dl
Glu	88 mg/dl	85 mg/dl		75-115 mg/dl
Cr	0.6 mg/dl	0.65 mg/dl		0.40-1.10 mg/dl
K^+^	4.11 mmol/L	3.95 mmol/L		3.5-5.1mmol/L
Na^+^	142.2 mmol/L	140.1 mmol/L		136-145 mmol/L
CEA	3.25 ng/mL			<5 ng/mL
CA125	95.8 U/mL			≤35 U/mL
CA15-3	17.1 U/mL			0.0-31.3 U/mL
CA19-9	15.4 U/mL			0.0-37 U/mL

**Figure 3:**
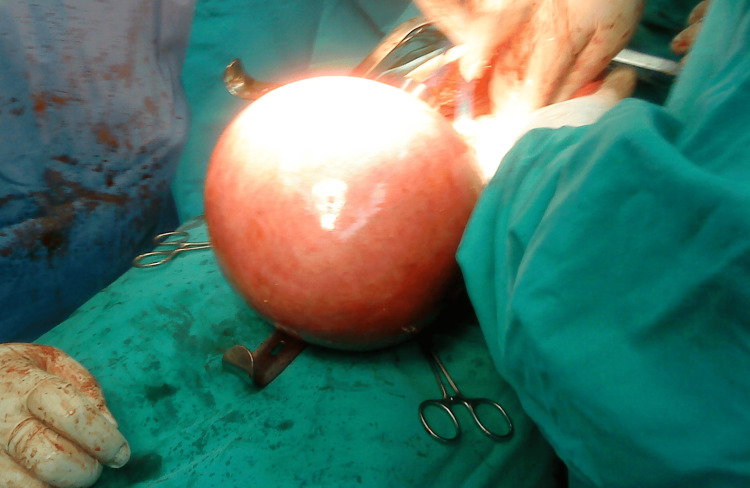
Intraoperative image of a giant uterine leiomyoma The presence of a massively enlarged leiomyomatous uterus is evident, for which an abdominal total hysterectomy with bilateral salpingectomy and oophorectomy was performed.

Based on clinical and imaging findings, the diagnosis of a myomatous uterus was confirmed, and a laparotomy was performed. Intraoperatively, after a midline vertical incision extended above the umbilicus, a giant uterine leiomyoma was identified (Figure 3).

An abdominal total hysterectomy with bilateral salpingectomy and oophorectomy was performed. The surgical challenges were numerous, and blood loss was significant (Table 1). Histological examination of the surgical specimen revealed that the uterus, with total dimensions of 35 × 26 × 22 cm and a weight of 9.8 kg, was significantly deformed, primarily due to the presence of a large myomatous tumor at the fundus with a maximum diameter of 25 cm. Two additional smaller tumors, measuring 6 cm and 7.5 cm, were located on the anterior and posterior uterine walls, respectively. No increased cellularity, necrosis, atypia, or elevated mitotic activity was observed in the tumor sections. After a five-day hospitalization and an uneventful postoperative course, the patient was discharged from the clinic.

## Discussion

Diagnosing large uterine leiomyomas is not easy. In some cases, patients may exhibit no symptoms or be unaware of the presence of a large uterine leiomyoma, with the diagnosis often made incidentally. Common symptoms of large leiomyomas include progressive abdominal enlargement, abnormal uterine bleeding, dysmenorrhea, constipation, urinary frequency, weight loss, back pain, pelvic pain, or pressure-related symptoms in the abdominal area [10,11]. Acute abdominal pain accompanied by nausea and vomiting in patients with giant uterine leiomyomas is an extremely rare clinical manifestation, potentially caused by uterine torsion and subsequent degenerative or necrotic changes in the tumor [12]. Other unusual clinical manifestations include acute edema, thrombosis, calcified pelvic masses, hematometra, severe pulmonary hypertension, and respiratory failure. These symptoms should not be overlooked or underestimated, as they may even pose a life-threatening risk to the patient [11]. A rare case recently described in the literature involved a patient with heart failure of unknown origin, which was attributed to the presence of a giant uterine leiomyoma [13]. In our patient, despite the presence of a large intrauterine mass weighing approximately 10 kg, there were no symptoms from the reproductive, respiratory, gastrointestinal, or urinary systems. The diagnosis of the giant uterine leiomyoma was made incidentally during the patient’s first visit to the gynecological clinic for a routine Pap smear. The large capacity of the peritoneal cavity, combined with its flexibility and the slow growth rate of benign uterine leiomyomas, explains their asymptomatic presence, even when these benign leiomyomatous tumors reach giant dimensions [14].

Modern imaging methods are crucial for the accurate preoperative diagnosis of giant uterine leiomyomas. Ultrasound, due to its high availability, noninvasive nature, and favorable cost-benefit ratio, remains the first-line imaging technique for the initial assessment of gynecological pathology. The diagnostic information provided by this method regarding confirmation of the clinical diagnosis and the extent of leiomyoma involvement is significant [15]. CT imaging can offer additional information about the number, size, location, and characteristics of leiomyomas. It can also detect potential complications, such as malignant transformation [16]. MRI, particularly in cases where leiomyomas have not undergone degeneration, is considered capable of preoperatively distinguishing benign uterine leiomyomas from leiomyosarcomas in most patients [17]. Additionally, the contribution of Fluorine-18 fluorodeoxyglucose PET/CT is considered significant today for the accurate diagnosis of giant uterine leiomyomas, especially in rare cases where other intra-abdominal pathologies coexist [18]. However, in patients with large pedunculated leiomyomas that have undergone hyaline degeneration, preoperative diagnosis is difficult. In such cases, differentiating a degenerated large uterine leiomyoma from malignant ovarian masses presents increased challenges, and misdiagnosis is common [19]. Other pathological conditions requiring differential diagnosis from large uterine masses include leiomyosarcoma, adenomyosis, carcinosarcoma, and endometrial stromal sarcoma [20]. In our patient, the preoperative diagnosis was based on clinical findings and CT imaging, as MRI was unavailable at our hospital, within a period of less than three months.

The need for treating giant uterine leiomyomas today is infrequent, as patients are often treated before the fibroids reach such giant dimensions. Factors such as fear of doctors and hospital visits, fear of surgical outcomes and potential failure, and religious beliefs about delaying fibroid treatment after prayers are the main reasons for delayed treatment of uterine leiomyomas, which can result in the development of giant tumors [21]. Surgical treatment, following appropriate preoperative preparation and multidisciplinary planning, remains the cornerstone of therapy for giant uterine leiomyomas. Total abdominal hysterectomy with or without bilateral salpingectomy and oophorectomy, depending on the patient's age and desire for fertility preservation, is the primary therapeutic choice for women who do not wish to retain fertility [22]. Similarly, preoperative embolization of the uterine artery, which can significantly reduce intraoperative blood loss, is a safe and effective option for the treatment of giant uterine fibroids. However, the disadvantages of this method include a higher risk of complications and reinterventions compared to non-giant leiomyomas [23]. In some patients with giant uterine leiomyomas who desire future pregnancies, open myomectomy can be proposed, although obtaining consent for the potential need for hysterectomy is essential [21]. Furthermore, the diagnosis and management of giant uterine leiomyomas during pregnancy, particularly in advanced stages, represent a significant challenge. Referral to a tertiary center with expertise in obstetric surgery and strict multidisciplinary planning are essential measures for the safe and successful management of pregnant patients [24]. In our patient, there were no symptoms related to the presence of the giant uterine leiomyoma. The decision to perform an abdominal total hysterectomy was based on the tumor’s extensive size, the deformed uterine anatomy, and the patient’s lack of desire for future pregnancies.

Most uterine leiomyomas are benign. Malignant transformation of uterine leiomyomas is a rare but serious complication, with an estimated incidence of 0.1-0.5% of all cases [25]. However, the morbidity and mortality rates for patients undergoing laparotomy to manage giant uterine leiomyomas are high [26]. Hemodynamic instability due to significant blood loss is the most common intraoperative complication [27]. Postoperative complications, such as venous thrombosis and acute renal failure, are also frequently observed [28].

## Conclusions

Giant uterine leiomyomas are rare, and their presence without symptoms is even rarer. Although giant uterine leiomyomas remain a diagnostic and surgical challenge, requiring multidisciplinary collaboration and management in specialized centers, in certain cases, such as our patient, they can be successfully treated without complications following accurate preoperative diagnosis and sufficient surgical expertise.
